# The role and mechanisms of gut microbiota in blood pressure regulation and cardiovascular health in hypertensive patients: an intervention study based on probiotics and high-fiber diets

**DOI:** 10.3389/fcvm.2026.1726604

**Published:** 2026-04-01

**Authors:** Chen Ming-Ming, Wu Shi-Ping, Xiong Yi-Ni, Fu Xue-Lian, Zhang Xiao-Tian

**Affiliations:** Department of Oncology, Suining Traditional Chinese Medicine Hospital, Suining City, Sichuan, China

**Keywords:** blood pressure, cardiovascular health, gut microbiota, high-fiber diets, hypertensive, probiotics

## Abstract

**Purpose:**

This study aimed to investigate the effects and mechanisms of probiotics combined with a high-fiber diet (PHFD) on blood pressure regulation and cardiovascular health in hypertensive patients.

**Methods:**

A retrospective cohort study was conducted involving 322 hypertensive patients admitted between January 2020 and June 2023. Patients were defined into two groups: conventional treatment (*n* = 186) and PHFD intervention (*n* = 136). The PHFD group received Bifidobacterium Triple Viable Capsules and a personalized high-fiber diet plan for three months. Data collected included demographic characteristics, blood pressure measurements, blood rheology indices, vascular endothelial function markers, inflammatory cytokine levels, gut microbiota composition, and SCFA concentrations. Blood samples and fecal samples were analyzed using biochemical assays, ELISA kits, automated biochemical analyzers, and advanced sequencing techniques.

**Results:**

After the intervention, the PHFD group showed significantly lower systolic BP (*P* = 0.006) and diastolic BP (*P* = 0.004) compared to the conventional group. The PHFD group also demonstrated a better therapeutic efficacy (*P* = 0.006). Significant improvements were observed in the PHFD group for erythrocyte aggregation index (*P* = 0.005), plasma fibrinogen levels (*P* = 0.010), ET-1 levels (*P* = 0.020), SOD levels (*P* = 0.045), and IL-6 levels (*P* = 0.014). Gut microbiota analysis revealed significant increases in beneficial bacterial families (Ruminococcaceae, Bifidobacteriaceae) and higher SCFA concentrations (acetate, propionate, butyrate) in the PHFD group (all *P* < 0.05).

**Conclusion:**

Probiotics and high-fiber diet interventions effectively modulate gut microbiota and SCFAs, resulting in significant improvements in blood pressure and cardiovascular health among hypertensive patients. These findings highlight the potential of integrated gut microbiota-targeted therapies as novel strategies for hypertension management.

## 1 Introduction

Hypertension remains a significant global health challenge, affecting over one billion individuals worldwide and contributing to substantial morbidity and mortality. It is a major risk factor for cardiovascular diseases (CVD), including coronary artery disease, heart failure, and stroke (1, 2). Despite the availability of various antihypertensive medications, achieving optimal blood pressure control remains elusive for many patients. This underscores the need for novel therapeutic approaches that can complement existing treatments. Recent advances in microbiome research have highlighted the critical role of gut microbiota in modulating systemic inflammation, immune function, and metabolic pathways, all of which are implicated in the pathophysiology of hypertension (3, 4).

The gut microbiota constitutes a complex ecosystem of microorganisms residing in the gastrointestinal tract, playing an essential role in host metabolism and homeostasis. Dysbiosis, or an imbalance in the gut microbial community, has been associated with numerous chronic conditions, including hypertension (5, 6). Studies have demonstrated that alterations in gut microbiota composition can lead to increased production of pro-inflammatory cytokines and reduced levels of beneficial short-chain fatty acids (SCFAs), such as acetate, propionate, and butyrate. These SCFAs are produced through the fermentation of dietary fibers by gut bacteria and are known to exert anti-inflammatory effects, improve endothelial function, and regulate blood pressure (7, 8). Consequently, interventions aimed at restoring gut microbial balance may offer a promising avenue for managing hypertension.

Probiotics and high-fiber diets represent two such interventions with potential benefits for hypertensive patients. Probiotics, defined as live microorganisms that confer health benefits when administered in adequate amounts, have been shown to enhance gut barrier integrity and reduce systemic inflammation. Commonly used probiotic strains include Lactobacillus and Bifidobacterium species, which are known to produce SCFAs and other bioactive metabolites (9, 10). High-fiber diets, rich in non-digestible carbohydrates, serve as substrates for beneficial gut bacteria, promoting their growth and activity. Together, these interventions can synergistically enhance the production of SCFAs and other metabolites, thereby improving gut health and potentially influencing blood pressure regulation (11, 12). However, the precise mechanisms by which these interventions affect gut microbiota and subsequently influence cardiovascular health remain incompletely understood.

Emerging evidence suggests that the gut microbiota plays a pivotal role in regulating blood pressure through multiple pathways. For instance, SCFAs produced by gut bacteria can activate specific receptors on vascular smooth muscle cells, leading to vasodilation and reduced blood pressure. Additionally, SCFAs have been shown to inhibit renin secretion from the kidneys, further contributing to blood pressure regulation. Moreover, gut microbiota-derived metabolites can modulate immune responses and reduce systemic inflammation, which is a key driver of endothelial dysfunction and hypertension (8, 13). Given these findings, it is plausible that interventions targeting gut microbiota could provide a holistic approach to managing hypertension and related cardiovascular complications.

Understanding the interplay between gut microbiota, dietary interventions, and cardiovascular health is crucial for developing effective strategies to manage hypertension. Few have systematically evaluated their combined effects on blood pressure and cardiovascular parameters in hypertensive patients. Furthermore, the mechanisms underlying these effects remain largely speculative, necessitating more comprehensive investigations. This study aims to address these gaps by investigating the effects of a combined probiotic and high-fiber diet intervention on gut microbiota composition, SCFA production, and cardiovascular health in hypertensive patients. Through this exploration, we seek to uncover novel insights into the complex interactions between gut microbiota, dietary factors, and blood pressure regulation, ultimately contributing to the development of innovative strategies for hypertension management.

## 2 Materials and methods

### 2.1 Study population and ethical considerations

This retrospective cohort study was approved by the Institutional Review Board and Ethics Committee of the Suining Traditional Chinese Medicine Hospital (Ethic Approval Number: 2023078). The requirement for informed consent was waived due to the use of exclusively de-identified historical patient data. We screened the medical records of hypertensive patients who were admitted to our hospital between January 2020 and June 2023.

The inclusion criteria were as follows: patients aged between 18 and 65 years, of any gender, diagnosed with primary hypertension at Grade 1 or 2 according to the 2010 Chinese Guidelines for the Management of Hypertension (14), and without severe hypertensive complications. Eligible patients also had no history of joint injury or regular exercise habits, and possessed complete medical records, follow-up data, and archived fecal and blood samples for subsequent analysis.

Patients were excluded from the analysis if they met any of the following criteria: a diagnosis of secondary hypertension; hypertension classified as Grade 3 or stratified as high/very high cardiovascular risk; the presence of severe hypertensive complications; a history of major surgery within the preceding six months; the use of antibiotics, prebiotics, probiotics, or regular intake of traditional Chinese medicine known to affect gut microbiota within three months before the study period; or a history of severe heart, liver, or renal failure, or malignancy. All procedures for biospecimen collection, processing, and storage complied with institutional biosafety and ethical standards.

### 2.2 Group allocation and intervention strategies

A total of 322 patients met the eligibility criteria and were included in the final analysis. Based on the documented intervention methods in their electronic medical records, patients were defined into two groups. Patients who received conventional antihypertensive treatment and standard lifestyle education were defined as the conventional group, consisting of 186 individuals. Patients who, in addition to conventional treatment and intervention, received a standardized “probiotics combined with high-fiber diet” regimen for three months, clearly prescribed by doctors and dietitians, were defined as the probiotics and high-fiber diet (PHFD) intervention group, totaling 136 individuals.

Patients in the conventional group received conventional antihypertensive pharmacotherapy and standard lifestyle education, which included generalized advice such as “low-salt, low-fat diet.” Their medical records contained no specific physician orders or follow-up documentation for the structured probiotic supplementation and high-fiber diet regimen prescribed to the PHFD group. Details of antihypertensive medication classes, dosages, and adherence patterns for both groups are summarized in Supplementary Table S1.

The PHFD group received a standardized intervention in addition to conventional antihypertensive treatment. The intervention was explicitly prescribed by physicians and dietitians for a duration of three months. The probiotic component involved the oral administration of Bifidobacterium Triple Viable Capsules (Manufacturer: Livzon Pharmaceutical Group Inc., Zhuhai, China; National Medicine Permit No.: S10960040) at a dose of 420 mg, twice daily. Adherence was verified through pharmacy dispensing records and patient self-reports that were subsequently confirmed by healthcare providers. The dietary component consisted of a personalized high-fiber diet plan. The dietary records explicitly guided patients to increase their intake of whole grains (e.g., oats, brown rice), legumes, vegetables, and fruits. To quantify adherence, dietary intake was assessed using records from a designated mobile application, where patients logged their daily food and beverage consumption via text, image, or voice. These records were processed using the professional nutrition analysis software Nutrium, which incorporates a Chinese food composition database, to precisely calculate the average daily dietary fiber intake for each patient during the intervention period. Patients included in the PHFD group were required to have an average daily fiber intake of ≥25 grams, confirming good adherence to the protocol. Adherence to the probiotic supplementation regimen was verified retrospectively using both pharmacies dispensing records and physician documentation in progress notes. For the dietary component, adherence was defined as achieving a mean daily fiber intake of ≥25 grams, consistent with recommendations by the Chinese Nutrition Society (2022). This threshold represents the minimum adequate intake for Chinese adults to support cardiometabolic health. Patients whose dietary logs indicated <25 g/day fiber intake or incomplete entries were excluded from the PHFD group to ensure adherence consistency.

### 2.3 Data acquisition and efficacy evaluation

Patient data were systematically extracted from the hospital's electronic medical record system. The collected data encompassed demographic characteristics, serial blood pressure measurements, therapeutic efficacy, blood rheology indices, vascular endothelial cell function markers, inflammatory cytokine levels, gut microbiota composition, and concentrations of gut microbiota-derived metabolite short-chain fatty acids (SCFAs).

Hypertension grading was defined based on the 2010 Chinese Guidelines for the Management of Hypertension (14). The grading was defined as follows: Grade 1 (Mild): systolic blood pressure (SBP) 140–159 mmHg and/or diastolic blood pressure (DBP) 90–99 mmHg; Grade 2 (Moderate): SBP 160–179 mmHg and/or DBP 100–109 mmHg; Grade 3 (Severe): SBP ≥180 mmHg and/or DBP ≥110 mmHg.

The primary outcome was therapeutic efficacy assessed after three months of intervention, which was categorized into three classes: markedly effective, effective, and ineffective. An outcome was considered markedly effective if the diastolic blood pressure decreased by more than 10 mmHg and returned to the normal range, or if it decreased by 20 mmHg even without normalization. An effective outcome was defined as diastolic pressure returning to normal without a 10 mmHg drop, a diastolic pressure reduction of 10–19 mmHg without normalization, or a systolic pressure reduction of at least 30 mmHg. Outcomes that did not meet these criteria were classified as ineffective.

### 2.4 Blood sample and biochemical assays

Following the intervention period, fasting venous blood samples were collected from all subjects for comprehensive analysis. Blood rheology indices, including whole blood viscosity at different shear rates, plasma viscosity, erythrocyte aggregation index, and plasma fibrinogen levels, were measured using a fully automated blood rheometer (LG-R-80B, Steellex, China).

The function of vascular endothelial cells was assessed by measuring the concentrations of endothelin-1 (ET-1), thrombomodulin (TM), vascular endothelial growth factor (VEGF), serum superoxide dismutase (SOD), and reactive oxygen species (ROS). These analyses were performed using specific commercial enzyme-linked immunosorbent assay (ELISA) kits (Human ET-1 ELISA Kit, Abcam, ab133030; Human Thrombomodulin ELISA Kit, Abcam, ab46508; Human VEGF ELISA Kit, Abcam, ab222510; Human SOD ELISA Kit, Cayman Chemical, 706002; ROS Assay Kit, Sigma-Aldrich, MAK143) according to the manufacturers' instructions. Absorbance was read on a Multiskan GO microplate reader (Thermo Fisher Scientific, USA).

Systemic inflammation was evaluated by quantifying the plasma levels of cytokines Interleukin-6 (IL-6), Interleukin-23 (IL-23), Interleukin-10 (IL-10), and Tumor Necrosis Factor-alpha (TNF-α). These analyses were conducted using an automated biochemical analyzer (Cobas c 501 module, Roche Diagnostics, Switzerland) with corresponding electrochemiluminescence immunoassay reagent kits (Roche Diagnostics).

### 2.5 Gut microbiota analysis

A total of 242 fecal samples (PHFD: *n* = 102; Conventional: *n* = 140) passed quality control and were included in the gut microbiota analysis. Patients with available samples did not differ significantly from the total cohort in terms of age, sex, BMI, or baseline blood pressure. This helped minimize the risk of sampling bias. Microbiota sequencing and downstream analysis were conducted blinded to group allocation. The composition of the gut microbiota was analyzed from archived fecal samples. Microbial genomic DNA was extracted, and the hypervariable V3–V4 region of the bacterial 16S rRNA gene was amplified by PCR. Sequencing was performed on the PacBio Sequel II platform (Pacific Biosciences, USA) to generate high-fidelity long reads. The resulting sequences were processed and analyzed using bioinformatics tools to determine microbial community structure, including alpha diversity indices (Shannon and Simpson), and relative taxonomic abundance at the phylum, genus, and species levels. Raw sequences from PacBio Sequel II were processed using the QIIME2 (v2022.2) pipeline. Denoising and chimera removal were performed using DADA2, and taxonomic classification was conducted against the SILVA 138 database**.** Samples were rarefied to a sequencing depth of 25,000 reads per sample to normalize diversity comparisons. Alpha diversity was assessed using Shannon and Simpson indices. Differential abundance analysis between groups was conducted using LEfSe (Linear Discriminant Analysis Effect Size) with an LDA threshold of 2.0 and FDR-corrected *P* < 0.05. Beta diversity was analyzed using Bray-Curtis dissimilarity and visualized via principal coordinate analysis (PCoA). Group differences in community structure were statistically tested using PERMANOVA (999 permutations). Spearman correlation analysis was used to assess associations between gut microbial taxa, SCFA concentrations, and blood pressure levels.

### 2.6 Quantification of short-chain fatty acids

The concentrations of SCFAs in plasma samples, including acetate, propionate, and butyrate, were determined before and after the intervention. Following collection in heparin sodium anticoagulant tubes and plasma separation, proteins were precipitated with methanol. The plasma samples were then acidified, and SCFAs were extracted and purified using solid-phase extraction columns with PAX as the sorbent. The analysis was conducted using an Agilent 8890 Gas Chromatograph system coupled with an Agilent 5977B Mass Spectrometric Detector (GC-MS) (Agilent Technologies, USA). The analytical method was fully validated for specificity, linearity, precision, accuracy, matrix effect, extraction recovery, and stability before being applied to the patient samples. SCFA concentrations were expressed in μmol/L. Plasma samples were processed immediately after collection: centrifuged, deproteinized with methanol, acidified with 0.15 M HCl, and stored at −80 °C until analysis. An internal standard (isocaproic acid) was used for quantification. GC-MS calibration curves were generated using SCFA standards (Sigma-Aldrich) in the range of 1–200 μmol/L. The validated method showed intra- and inter-assay CVs <10%. All samples were analyzed in duplicate.

### 2.7 Statistical analysis

Statistical analysis was performed using SPSS 29.0 (SPSS Inc., Chicago, IL, USA). Categorical variables are expressed as frequencies and proportions [*n* (%)]. Chi-square tests were utilized for categorical data. Continuous variables were assessed for normality using the Shapiro–Wilk test. Variables that conformed to a normal distribution are reported as means ± standard deviations (M ± SD). The threshold for statistical significance was set at *P* < 0.05.

## 3 Results

### 3.1 Baseline demographic and clinical characteristics

There were no significant differences in gender distribution, age, BMI, hypertension duration, diabetes prevalence, smoking history, drinking history, or hypertension grade (all *P* > 0.05) (Table 1). Laboratory measurements, including fasting blood glucose, HbA1c, total cholesterol, and triglycerides, also did not differ significantly between the groups (all *P* > 0.05).

**Table 1:** Comparison of baseline demographic and clinical characteristics between the two groups.

Parameters	Conventional group (*n* = 186)	PHFD intervention group (*n* = 136)	*t*/*χ*^2^	*P*
Gender [*n* (%)]			0.349	0.555
Female	80 (43.01%)	63 (46.32%)		
Male	106 (56.99%)	73 (53.68%)		
Age (years)	54.75 ± 4.53	55.17 ± 4.64	0.803	0.423
BMI (kg/m2)	24.05 ± 1.46	24.11 ± 1.38	0.388	0.698
Hypertension duration (years)	9.43 ± 2.85	9.73 ± 2.52	0.956	0.340
Diabetes [*n* (%)]	32 (17.2%)/154 (82.8%)	21 (15.44%)/115 (84.56%)	0.178	0.673
Smoking history [*n* (%)]	55 (29.57%)/131 (70.43%)	36 (26.47%)/100 (73.53%)	0.372	0.542
Drinking history [*n* (%)]	48 (25.81%)/138 (74.19%)	40 (29.41%)/96 (70.59%)	0.514	0.473
Hypertension grade [*n* (%)]			0.086	0.769
Grade 1	105 (56.45%)	79 (58.09%)		
Grade 2	81 (43.55%)	57 (41.91%)		
Fasting blood glucose (mmol/L)	5.82 ± 0.71	5.79 ± 0.68	0.305	0.761
HbA1c (%)	6.12 ± 0.51	6.07 ± 0.43	0.821	0.412
Total cholesterol (mmol/L)	5.05 ± 0.89	4.98 ± 0.92	0.672	0.502
Triglycerides (mmol/L)	1.75 ± 0.43	1.72 ± 0.41	0.676	0.499

PHFD, probiotics and high-fiber diet; BMI, Body Mass Index; HbA1c, Glycated Hemoglobin.

### 3.2 Blood pressure changes and therapeutic efficacy

**Figure 1:**
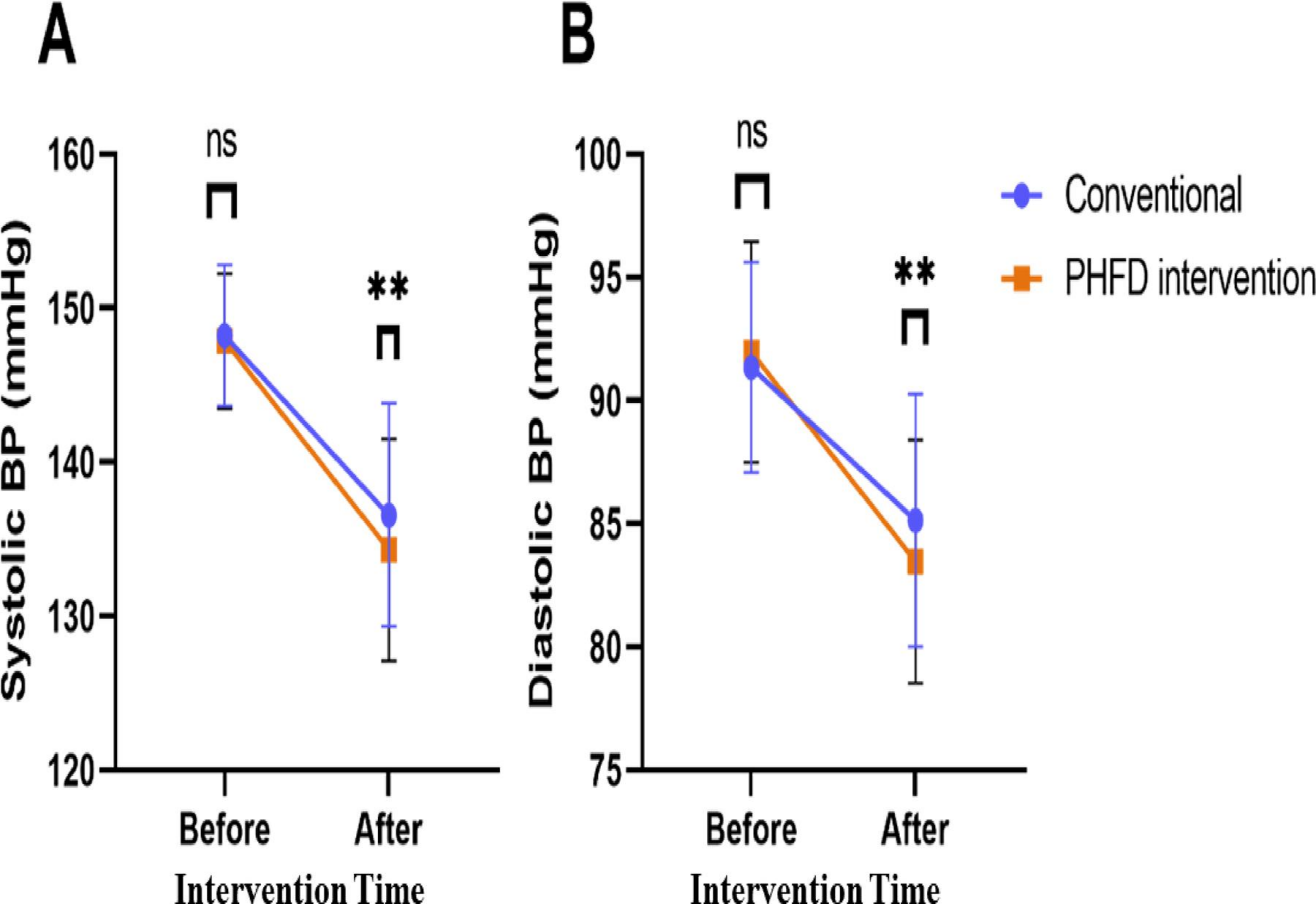
Comparison of blood pressure between two groups. **(A)** Systolic BP; **(B)** Diastolic BP. PHFD, probiotics and high-fiber diet; BP, blood pressure; ns, no significant difference; **P < 0.01.

Before intervention, there were no significant differences in systolic BP or diastolic BP between the two groups (*P* > 0.05) (Figure 1). After intervention, the PHFD intervention group showed significantly lower systolic BP (134.31 ± 7.22 vs. 136.55 ± 7.25, *t* = 2.751, *P* = 0.006) and diastolic BP (83.47 ± 4.94 vs. 85.13 ± 5.13, *t* = 2.908, *P* = 0.004).

The comparison of therapeutic efficacy between the conventional group and the PHFD intervention group revealed significant differences in the distribution of outcomes (*χ*^2^ = 10.133, *P* = 0.006) (Table 2). The PHFD intervention group showed a significantly higher rate of markedly effective responses (40.44% vs. 24.19%). The proportions of effective responses were similar between the two groups (45.59% vs. 54.84%), and the rate of ineffective responses was lower in the PHFD intervention group (13.97% vs. 20.97%). To adjust for potential confounding variables, multivariate regression models were applied for key outcomes, as detailed in Supplementary Table S2.

**Table 2:** Comparison of therapeutic efficacy between the two groups.

Parameters	Conventional group (*n* = 186)	PHFD intervention group (*n* = 136)	*χ* ^2^	*P*
			10.133	0.006
Markedly effective	45 (24.19%)	55 (40.44%)		
Effective	102 (54.84%)	62 (45.59%)		
Ineffective	39 (20.97%)	19 (13.97%)		

### 3.3 Blood rheology indices

Whole blood viscosity at high-shear and low-shear conditions, along with plasma viscosity, did not differ significantly between the groups (all *P* > 0.05) (Table 3). In contrast, the PHFD intervention group had a significantly lower erythrocyte aggregation index (4.02 ± 0.41 vs. 4.15 ± 0.45, *t* = 2.818, *P* = 0.005) and plasma fibrinogen levels (3.35 ± 0.35 vs. 3.45 ± 0.38, *t* = 2.580, *P* = 0.010).

**Table 3:** Comparison of blood rheology indices between the two groups.

Parameters	Conventional group (*n* = 186)	PHFD intervention group (*n* = 136)	*t*	*P*
Whole Blood Viscosity (mPa·s, High-shear)	5.52 ± 0.91	5.45 ± 0.89	0.673	0.502
Whole Blood Viscosity (mPa·s, Low-shear)	8.65 ± 1.32	8.50 ± 1.35	0.938	0.349
Plasma Viscosity (mPa·s)	1.61 ± 0.15	1.59 ± 0.14	0.942	0.347
Erythrocyte Aggregation Index	4.15 ± 0.45	4.02 ± 0.41	2.818	0.005
Plasma Fibrinogen (g/L)	3.45 ± 0.38	3.35 ± 0.35	2.580	0.010

PHFD, probiotics and a high-fiber diet.

### 3.4 Vascular endothelial cell function

For ET-1, the PHFD intervention group had significantly lower levels compared to the conventional group (62.26 ± 7.52 vs. 64.33 ± 8.15, *t* = 2.332, *P* = 0.020) (Table 4). Similarly, SOD levels were significantly higher in the PHFD intervention group (138.82 ± 16.57 vs. 135.24 ± 15.15, *t* = 2.013, *P* = 0.045). There were no significant differences observed for TM, VEGF, or ROS levels between the two groups (all *P* > 0.05).

**Table 4:** Comparison of vascular endothelial cell function markers between two groups.

Parameters	Conventional group (*n* = 186)	PHFD intervention group (*n* = 136)	*t*	*P*
ET-1 (pg/mL)	64.33 ± 8.15	62.26 ± 7.52	2.332	0.020
TM (ng/mL)	8.56 ± 1.22	8.38 ± 1.15	1.359	0.175
VEGF (pg/mL)	185.57 ± 25.31	189.65 ± 26.46	1.400	0.162
SOD (U/mL)	135.24 ± 15.15	138.82 ± 16.57	2.013	0.045
ROS (U/mL)	350.52 ± 42.86	342.37 ± 41.03	1.715	0.087

PHFD, probiotics and high-fiber diet; ET-1, ENDOTHELIN-1; TM, thrombomodulin; VEGF, vascular endothelial growth factor; SOD, superoxide dismutase; ROS, reactive oxygen species.

### 3.5 Inflammatory cytokine

The PHFD intervention group had significantly lower levels of IL-6 compared to the conventional group (21.5 ± 3.57 vs. 22.53 ± 3.81, *t* = 2.463, *P* = 0.014) (Table 5). There were no significant differences observed for IL-23, IL-10, or TNF-α between the two groups (all *P* > 0.05).

**Table 5:** Comparison of inflammatory cytokine levels between two groups (pg/mL).

Parameters	Conventional group (*n* = 186)	PHFD intervention group (*n* = 136)	*t*	*P*
IL-6	22.53 ± 3.81	21.5 ± 3.57	2.463	0.014
IL-23	155.35 ± 30.52	149.77 ± 29.83	1.636	0.103
IL-10	11.13 ± 2.25	11.53 ± 2.35	1.534	0.126
TNF-α	35.84 ± 5.18	35.15 ± 5.03	1.196	0.233

PHFD, probiotics and high-fiber diet; IL, interleukin; TNF-α, tumor necrosis factor-alpha.

### 3.6 Gut microbiota composition

**Figure 2:**
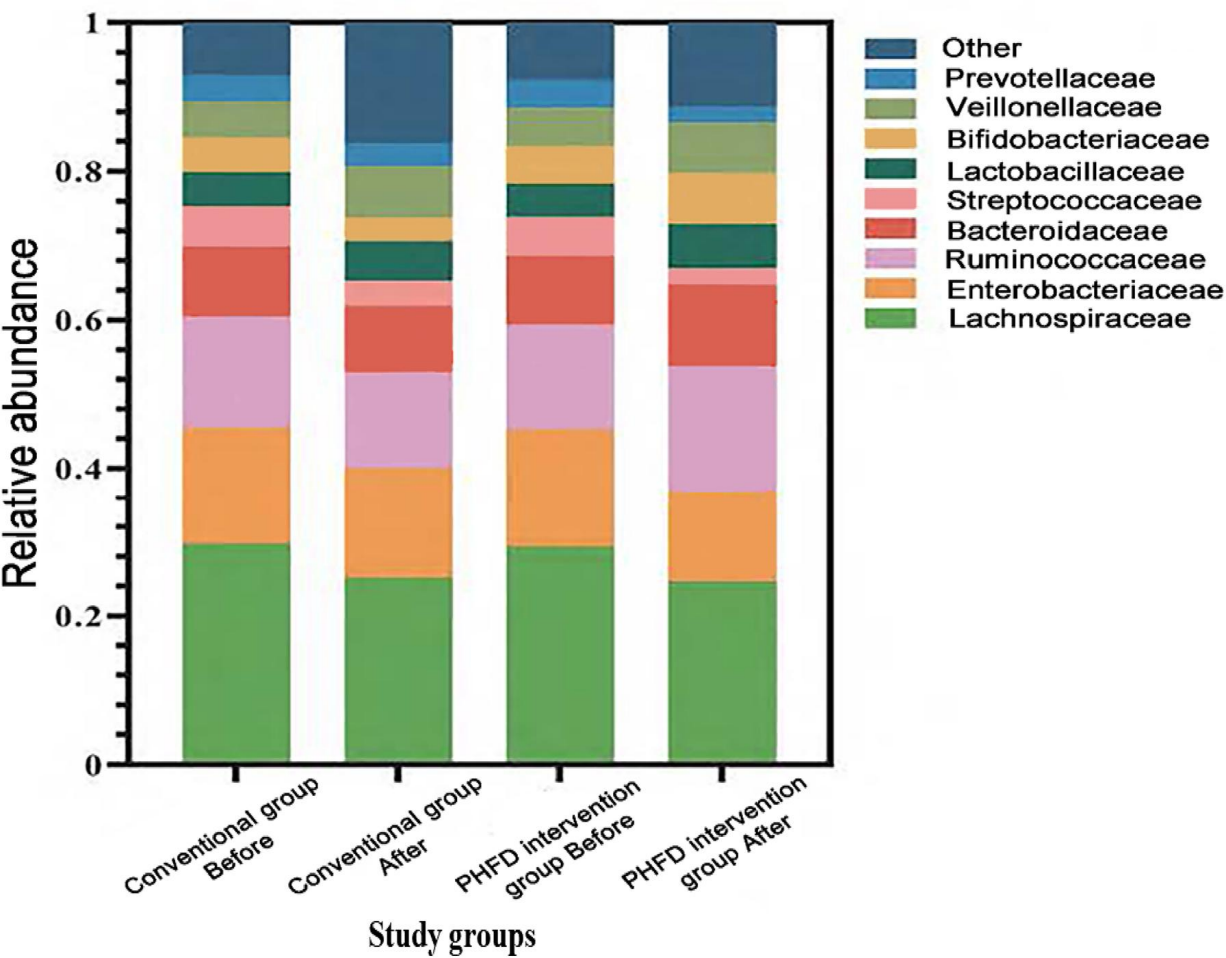
Comparison of gut microbiota composition between two groups. PHFD, probiotics and a high-fiber diet.

Of the total study population, gut microbiota sequencing was performed on fecal samples from 102 PHFD and 140 conventional group patients. Figure 2 illustrates the distinct shifts in microbial community structure between the two groups. The relative abundance of Lachnospiraceae showed a modest decline in both groups; however, this family includes both beneficial and neutral taxa, and its clinical significance remains uncertain without genus-level resolution. Notably, the relative abundances of several potentially harmful bacterial families, including Enterobacteriaceae, Streptococcaceae, and Prevotellaceae, decreased in both groups after treatment. However, this reduction was significantly more marked in the PHFD intervention group. Conversely, the intervention promoted the growth of beneficial bacterial families. Specifically, the PHFD group exhibited significant increases in the relative abundances of Ruminococcaceae, Bifidobacteriaceae, Lactobacillaceae, Bacteroidaceae, and Veillonellaceae compared to the conventional group. Beta-diversity analysis based on Bray-Curtis dissimilarity revealed distinct microbial community structures between groups (PERMANOVA, *P* = 0.001), shown in Supplementary Figure S1. LEfSe analysis identified several taxa significantly enriched in the PHFD group, including Ruminococcaceae, Bifidobacteriaceae, and Lactobacillaceae (LDA >2.0, FDR <0.05; Supplementary Figure S2). Additionally, Spearman correlation analysis revealed that the relative abundance of these SCFA-producing bacteria was positively correlated with SCFA levels and negatively correlated with both systolic and diastolic BP (*ρ* range: ±0.40–0.65, *P* < 0.01), detailed in Supplementary Table S3.

Before the intervention, there were no significant differences in the Shannon index (4.57 ± 0.22 vs. 4.53 ± 0.24, *t* = 1.515, *P* > 0.05) or Simpson index (0.84 ± 0.13 vs. 0.83 ± 0.15, *t* = 0.676, *P* > 0.05) between the two groups (Table 6). However, after the intervention, the PHFD intervention group showed significantly higher Shannon index (4.82 ± 0.28 vs. 4.52 ± 0.25, *t* = 9.782, *P* < 0.001) and lower Simpson index (0.68 ± 0.16 vs. 0.75 ± 0.12, *t* = 4.486, *P* < 0.001).

**Table 6:** Comparison of gut microbiota alpha diversity between two groups.

Parameters	Conventional group (*n* = 186)	PHFD intervention group (*n* = 136)	*t*	*P*
Before intervention				
Shannon index	4.57 ± 0.22	4.53 ± 0.24	1.515	0.131
Simpson index	0.84 ± 0.13	0.83 ± 0.15	0.676	0.500
After intervention				
Shannon index	4.52 ± 0.25	4.82 ± 0.28	9.782	<0.001
Simpson index	0.75 ± 0.12	0.68 ± 0.16	4.486	<0.001

PHFD, probiotics and a high-fiber diet.

### 3.7 Concentrations of gut microbiota-derived metabolite short-chain fatty acids

The comparison of SCFAs between the conventional group and the PHFD intervention group revealed significant differences in acetate, propionate, and butyrate levels (Table 7). The PHFD intervention group showed significantly higher levels of acetate (95.65 ± 15.83 vs. 85.33 ± 12.52, *t* = 6.298, *P* < 0.001), propionate (5.21 ± 1.42 vs. 4.76 ± 1.14, *t* = 3.031, *P* = 0.003), and butyrate (3.62 ± 1.04 vs. 3.31 ± 0.86, *t* = 2.844, *P* = 0.005) compared to the conventional group.

**Table 7:** Comparison of SCFAs between two groups.

Parameters	Conventional group (*n* = 186)	PHFD intervention group (*n* = 136)	*t*	*P*
Acetate	85.33 ± 12.52	95.65 ± 15.83	6.298	<0.001
Propionate	4.76 ± 1.14	5.21 ± 1.42	3.031	0.003
Butyrate	3.31 ± 0.86	3.62 ± 1.04	2.844	0.005

PHFD, probiotics and high-fiber diet; SCFAs, short-chain fatty acids.

## 4 Discussion

The current study aimed to investigate the role and mechanisms of gut microbiota in blood pressure regulation and cardiovascular health among hypertensive patients through a dietary intervention involving probiotics and a high-fiber diet. This section delves into potential explanations for observed outcomes, highlighting how alterations in gut microbial composition and increased production of SCFAs may contribute to improvements in blood pressure control and vascular endothelial function.

The PHFD intervention group demonstrated improved blood pressure levels compared to the conventional treatment group. These associations may be explained by previously described mechanisms in the literature. For instance, SCFAs have been shown to activate GPR41 and GPR43 receptors, promote vasodilation, and modulate immune cell activity, including Treg induction. While these pathways were not directly measured in this study, they represent plausible biological explanations supported by prior research. This enhanced barrier function reduces the translocation of endotoxins into the bloodstream, thereby decreasing systemic inflammation and lowering blood pressure (15, 16). Additionally, dietary fibers serve as prebiotics that promote the growth of beneficial bacteria capable of producing SCFAs, which activate G-protein coupled receptors (GPRs) like GPR41 and GPR43. Activation of these receptors leads to vasodilation, thus reducing systemic blood pressure (17, 18).

Despite no notable differences in whole blood viscosity or plasma viscosity between groups, the PHFD intervention group exhibited lower erythrocyte aggregation index and plasma fibrinogen levels. These findings suggest that the intervention positively impacted blood rheology, potentially via anti-inflammatory actions mediated by gut microbiota and their metabolites. SCFAs produced by gut microbiota can inhibit nuclear factor kappa-light-chain-enhancer of activated B cells (NF-κB) signaling, thereby reducing inflammation and subsequently decreasing fibrinogen synthesis (19). Furthermore, probiotics have been shown to improve endothelial function by enhancing nitric oxide (NO) production, which is critical for vasodilation and reduction in blood viscosity (20). High-fiber diets also contribute to this effect by promoting the growth of bacteria that produce SCFAs, which in turn modulate vascular tone and improve blood flow (21).

Improvements in endothelial cell function within the PHFD intervention group were evident through decreased ET-1 levels and increased SOD activity. ET-1 is a potent vasoconstrictor whose downregulation can enhance vasodilation. The observed increase in SOD activity suggests an enhanced antioxidant capacity, which could mitigate oxidative stress-induced damage to endothelial cells. Probiotics play a crucial role here by modulating the production of bioactive molecules that regulate these processes (10). For example, certain strains of Lactobacillus and Bifidobacterium have been shown to upregulate NO synthase expression, leading to increase NO production and subsequent vasodilation (22). Additionally, high-fiber diets promote the growth of bacteria that produce SCFAs, which can inhibit oxidative stress pathways and reduce endothelial dysfunction (23, 24).

Lower interleukin-6 (IL-6) levels in the PHFD intervention group point towards reduced systemic inflammation. Microbial dysbiosis has been linked with elevated pro-inflammatory cytokine production, whereas balanced gut microbiota can promote anti-inflammatory responses. SCFAs act as histone deacetylase inhibitors, influencing gene expression patterns associated with inflammation resolution (18, 25). Probiotics also exert anti-inflammatory effects by enhancing the production of regulatory T cells (Tregs), which suppress inflammatory responses. Lactobacillus and Bifidobacterium species have been shown to induce Treg differentiation, thereby reducing pro-inflammatory cytokine levels (26). High-fiber diets support these effects by providing substrates for SCFA-producing bacteria, further enhancing anti-inflammatory responses (27).

Alterations in gut microbiota composition following the PHFD intervention provide insights into the underlying mechanisms driving the clinical benefits observed. An increase in beneficial bacterial families like Ruminococcaceae, Bifidobacteriaceae, and Lactobacillaceae indicates a healthier gut ecosystem capable of producing higher levels of SCFAs. These bacteria ferment dietary fibers to produce SCFAs, which not only serve as energy sources for colonic epithelial cells but also exert systemic effects, including immune modulation and regulation of blood pressure. Furthermore, the reduction in potentially harmful bacterial families underscores the importance of maintaining a diverse and balanced gut microbiota for optimal health (28–30).

Elevated acetate, propionate, and butyrate concentrations in the PHFD intervention group correlate with improved cardiovascular health parameters. Each SCFA exerts unique physiological effects: acetate influences appetite regulation and lipid metabolism; propionate acts on immune cells to suppress inflammation; and butyrate supports colonic health and reduces oxidative stress. Together, these SCFAs create a favorable environment conducive to lowering blood pressure and enhancing endothelial function (31, 32). Acetate, by influencing adipocyte function, can reduce lipolysis and free fatty acid release, thereby improving insulin sensitivity and glucose metabolism (33). Propionate's ability to activate free fatty acid receptor 2 (FFAR2) and FFAR3 leads to enhanced glucagon-like peptide-1 (GLP-1) secretion, which improves insulin secretion and glycemic control (34). Butyrate, through its inhibition of histone deacetylases, promotes anti-inflammatory gene expression and reduces oxidative stress, thereby protecting against endothelial dysfunction (35). These mechanisms collectively contribute to the observed improvements in cardiovascular health among patients receiving the PHFD intervention.

This study has several limitations, including its reliance on observational data from electronic medical records, which may introduce biases. Additionally, while the intervention period was sufficient to detect changes, longer follow-up periods would provide more insight into the sustainability of these effects. Future research should aim to elucidate the precise molecular pathways through which gut microbiota and SCFAs exert their cardiovascular benefits. Moreover, randomized controlled trials with larger sample sizes are needed to confirm these findings and explore the potential for personalized therapeutic approaches targeting the gut microbiome in hypertension management. Adherence assessment relied on existing logs and retrospective review of app-based dietary records, which may not capture real-time compliance with complete accuracy. Patients with <25 g/day average fiber intake or incomplete diet logs were excluded from the PHFD group, ensuring consistent adherence within the intervention group. Furthermore, due to the retrospective cohort design and lack of randomization, the study is inherently subject to potential selection bias and treatment allocation bias. As a result, the findings reported should be interpreted as associations rather than causal relationships. Although we observed significant differences between groups, we cannot conclusively attribute these outcomes solely to the probiotic and high-fiber diet intervention. Future prospective randomized controlled trials are necessary to establish causality and eliminate potential confounding.

## 5 Conclusion

This study suggests that an intervention combining probiotics and a high-fiber diet may have the potential to positively influence blood pressure regulation and cardiovascular health in hypertensive patients by modulating gut microbiota composition and increasing SCFA production. The findings indicate that such an intervention could promote the growth of beneficial bacterial families, which may contribute to improved vascular endothelial function and reduced systemic inflammation. Additionally, the observed changes in gut microbiota and SCFAs suggest potential mechanisms through which this intervention might exert its effects, including modulation of erythrocyte aggregation and plasma fibrinogen levels. However, further longitudinal studies are necessary to confirm these preliminary observations and to elucidate the long-term efficacy and safety of this approach. Overall, while the results are promising, they highlight the need for additional research to fully understand the complex interactions between gut microbiota, dietary interventions, and cardiovascular outcomes in hypertensive populations. Moreover, mechanistic pathways such as GPR signaling, nitric oxide modulation, and immune regulation were discussed based on literature but were not directly investigated in this study. These interpretations should therefore be viewed as speculative and hypothesis-generating, warranting further molecular studies for confirmation.

## Data Availability

The datasets generated and/or analyzed during the current study are available from the corresponding author on reasonable request.
