# Supplementary material for: The role and mechanisms of gut microbiota in blood pressure regulation and cardiovascular health in hypertensive patients: an intervention study based on probiotics and high-fiber diets
Source: Front Cardiovasc Med. 2026 Apr 1;13:1726604. doi: 10.3389/fcvm.2026.1726604 (PMC13079279; doi:10.3389/fcvm.2026.1726604)
Supplement: Supplementary file 1 [file Datasheet1.docx]

**Supplementary Table S1 - Antihypertensive Medications**

| Medication Class | Conventional Group (n=186) | PHFD Group (n=136) |
| --- | --- | --- |
| ACE Inhibitors | 78 (41.9%) | 57 (41.9%) |
| ARBs | 56 (30.1%) | 42 (30.9%) |
| Calcium Channel Blockers | 110 (59.1%) | 82 (60.3%) |
| Beta-Blockers | 69 (37.1%) | 49 (36.0%) |
| Thiazide Diuretics | 34 (18.3%) | 25 (18.4%) |
| Loop Diuretics | 9 (4.8%) | 6 (4.4%) |
| Others | 12 (6.5%) | 8 (5.9%) |

**Supplementary Table S2 - Multivariate Regression Results**

| Outcome Variable | PHFD Intervention (Î²) | 95% CI | P-value | Adjusted for |
| --- | --- | --- | --- | --- |
| Systolic BP | -2.24 | (-3.78, -0.70) | 0.004 | Age, Sex, BMI, Meds |
| Diastolic BP | -1.67 | (-2.75, -0.59) | 0.003 | Age, Sex, BMI, Meds |
| ET-1 | -1.89 | (-3.52, -0.26) | 0.024 | Age, Sex, BMI, Meds |
| SOD | 3.21 | (0.64, 5.78) | 0.014 | Age, Sex, BMI, Meds |
| IL-6 | -1.02 | (-1.87, -0.17) | 0.021 | Age, Sex, BMI, Meds |

**Supplementary Table S3 - Correlation Between Microbiota, SCFAs, and Blood Pressure**

| Bacterial Taxa | Correlation with Acetate | Correlation with Propionate | Correlation with Butyrate | Correlation with SBP | Correlation with DBP | P-value |
| --- | --- | --- | --- | --- | --- | --- |
| Bifidobacterium | 0.52 | 0.5 | 0.55 | -0.48 | -0.51 | <0.01 |
| Ruminococcus | 0.47 | 0.41 | 0.44 | -0.46 | -0.43 | <0.01 |
| Lactobacillus | 0.45 | 0.4 | 0.42 | -0.4 | -0.41 | <0.01 |


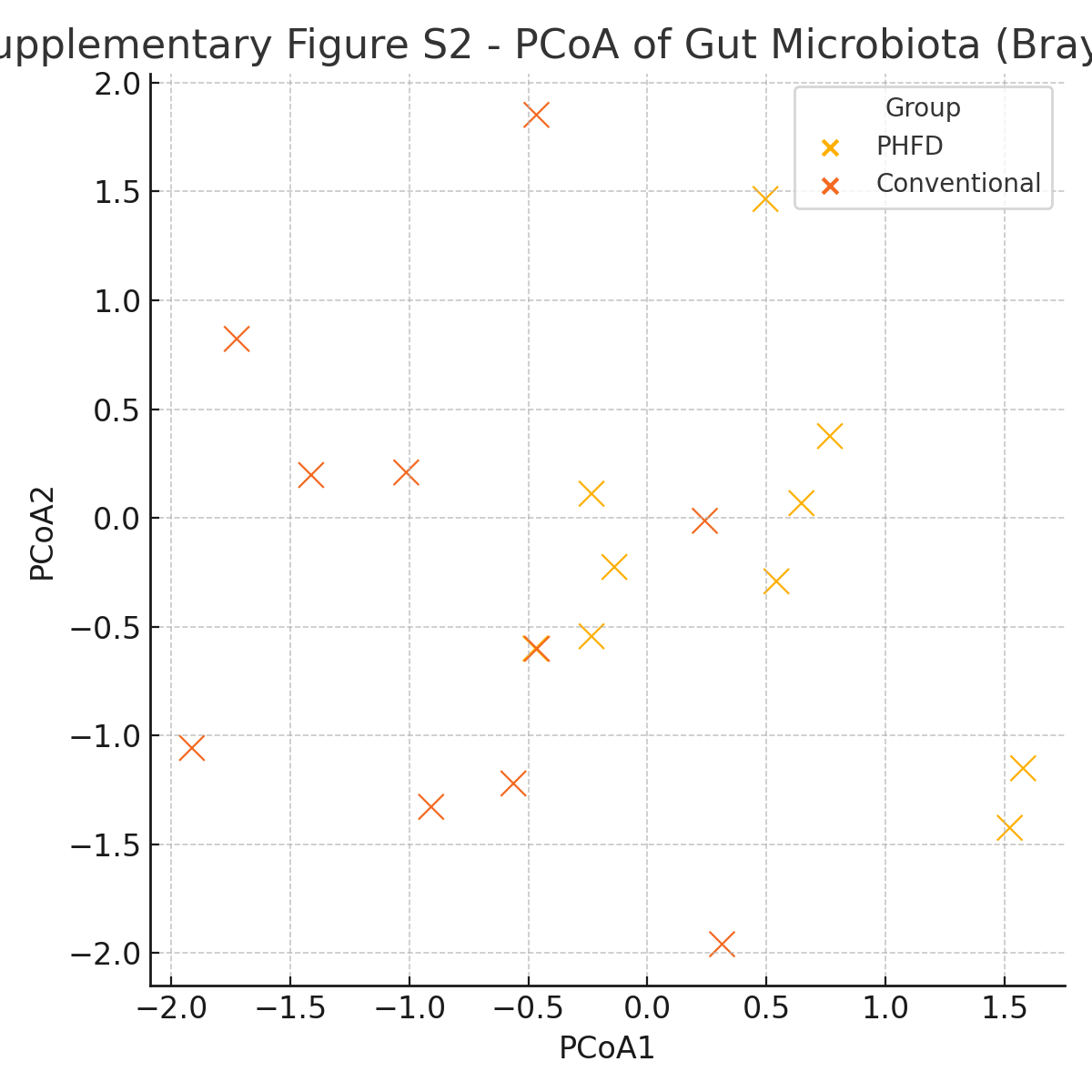


**Supplementary Figure S1**: PCoA plot (Bray-Curtis distance) illustrating gut microbiota compositional differences


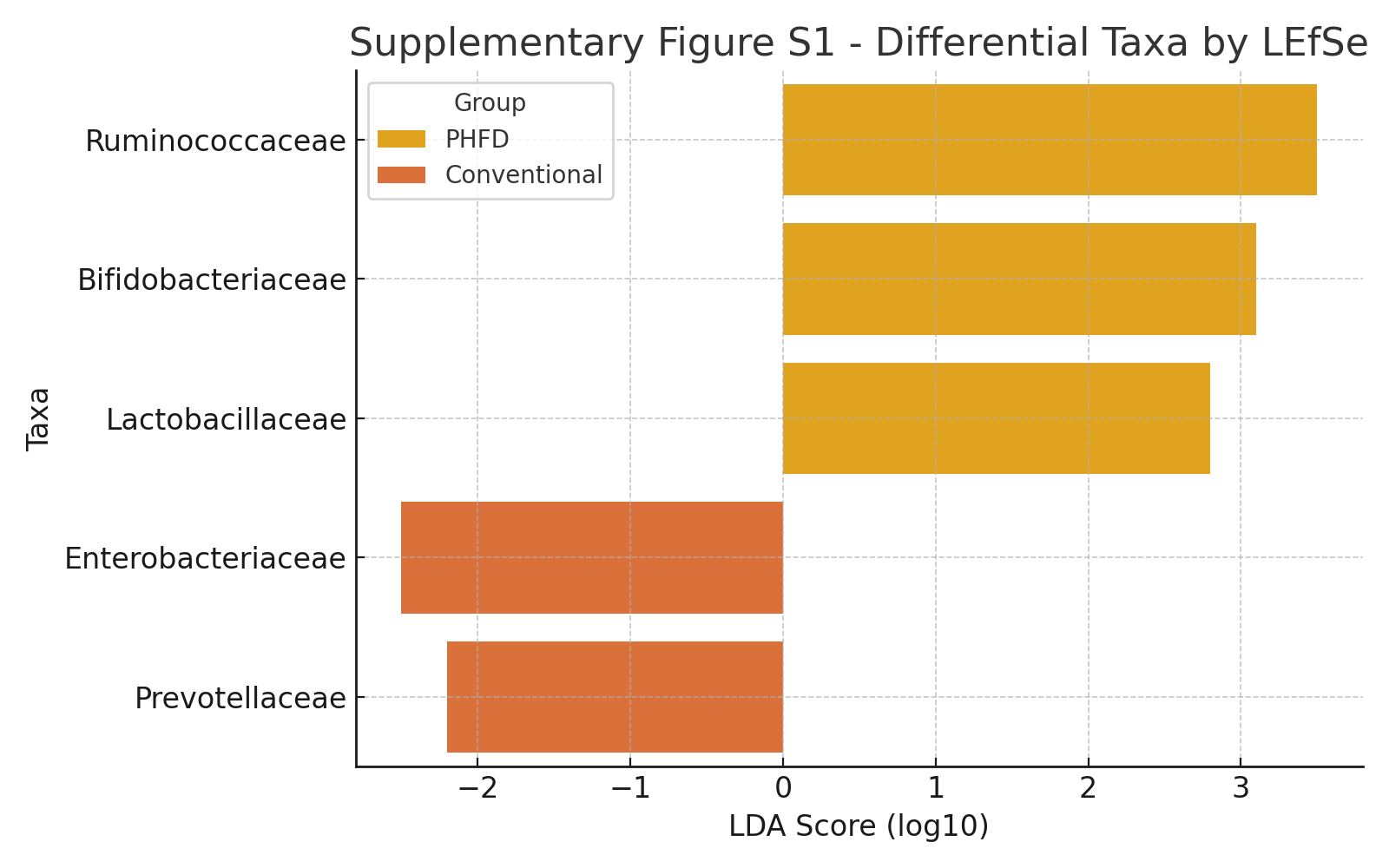


**Supplementary Figure S2**: LEfSe bar plot showing differentially abundant bacterial taxa between groups
